# Endothelial PRMT5 plays a crucial role in angiogenesis after acute ischemic injury

**DOI:** 10.1172/jci.insight.152481

**Published:** 2022-05-09

**Authors:** Qing Ye, Jian Zhang, Chen Zhang, Bing Yi, Kyosuke Kazama, Wennan Liu, Xiaobo Sun, Yan Liu, Jianxin Sun

**Affiliations:** 1 Department of Clinical Pharmacy, Xinhua Hospital, Shanghai Jiao Tong University School of Medicine, Shanghai, China.; 2 Center for Translational Medicine, Thomas Jefferson University, Philadelphia, Pennsylvania, USA.

**Keywords:** Angiogenesis, Vascular Biology, Hypoxia, Signal transduction

## Abstract

Arginine methylation mediated by protein arginine methyltransferases (PRMTs) has been shown to be an important posttranslational mechanism involved in various biological processes. Herein, we sought to investigate whether PRMT5, a major type II enzyme, is involved in pathological angiogenesis and, if so, to elucidate the molecular mechanism involved. Our results show that PRMT5 expression is significantly upregulated in ischemic tissues and hypoxic endothelial cells (ECs). Endothelial-specific *Prmt5*-KO mice were generated to define the role of PRMT5 in hindlimb ischemia–induced angiogenesis. We found that these mice exhibited impaired recovery of blood perfusion and motor function of the lower limbs, an impairment that was accompanied by decreased vascular density and increased necrosis as compared with their WT littermates. Furthermore, both pharmacological and genetic inhibition of PRMT5 significantly attenuated EC proliferation, migration, tube formation, and aortic ring sprouting. Mechanistically, we showed that inhibition of PRMT5 markedly attenuated hypoxia-induced factor 1-α (HIF-1α) protein stability and vascular endothelial growth factor–induced (VEGF-induced) signaling pathways in ECs. Our results provide compelling evidence demonstrating a crucial role of PRMT5 in hypoxia-induced angiogenesis and suggest that inhibition of PRMT5 may provide novel therapeutic strategies for the treatment of abnormal angiogenesis-related diseases, such as cancer and diabetic retinopathy.

## Graphical abstract

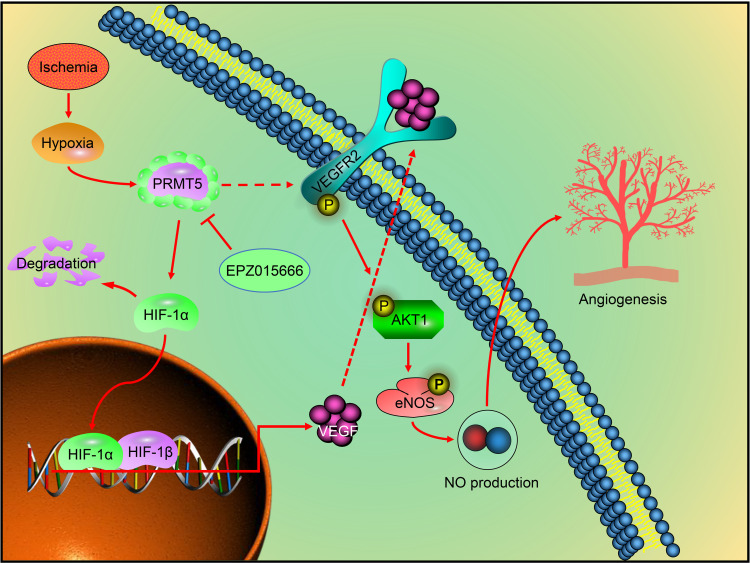

## Introduction

Angiogenesis is the process of capillary formation from preexisting blood vessels, and it is important for normal development, tissue homeostasis, and various physiological and pathological processes (1, 2). Under physiological conditions, pro- and antiangiogenic factors maintain a dynamic balance. Disruption of this balance can lead to abnormal angiogenesis, resulting in development of a variety of diseases, including cancer, diabetic retinopathy, vascular malformations, and delayed wound healing (3, 4). Angiogenesis is mainly initiated by hypoxia in poorly perfused tissues (5). Under normoxia, hypoxia-induced factor 1-α (HIF-1α) are hydroxylated by prolyl hydroxylase domain enzymes (PHDs) and factor-inhibiting HIF (FIH) in oxygen in a 2-oxoglutarate–dependent manner, followed by binding to the von Hippel–Lindau (VHL) E3 ubiquitin ligase complex, which consequently results in the degradation of HIF-1α by proteasome. However, in the presence of insufficient oxygen, PHDs and FIH are inactive, leading to stabilization of HIF-1α and formation of heterodimers with HIF-1β in the nucleus, where HIF-1 recruits transcriptional coactivator complexes and binds to hypoxia response elements (HRE) to activate transcription of target genes in angiogenesis (6–8). HIF-1α activity is also tightly regulated by various posttranslational modifications, such as phosphorylation, acetylation, and SUMOylation (9–11). Whether protein arginine methylation plays a key role in regulating HIF-1α activity remains largely unknown.

Protein arginine methyltransferases (PRMTs) have been characterized as critical regulators of cell homeostasis and are essentially involved in several biological processes, including RNA regulation, signal transduction, and chromatin regulation (12, 13). PRMT5 is the main type II PRMT, and it exerts multiple biological functions by catalyzing its histone or nonhistone substrates to form monomethyl arginine (MMA) and symmetric dimethyl arginine (SDMA) (13–15). PRMT5 is ubiquitously expressed with the higher expression in embryonic tissues (16). In addition, PRMT5 is essential for normal development, loss of which leads to prenatal death (17). Besides its importance in embryonic development, PRMT5 is also highly expressed in various cancer models, including leukemia, lymphoma, glioblastoma, lung cancer, and breast cancer (18, 19). Consistently, higher expression levels of PRMT5 are predicted with poor clinical outcome (20, 21). Indeed, preclinical studies have shown the potential for pharmacologic inhibition of PRMT5 in the treatment of cancer (22). EPZ015666 (GSK3235025) is a potent selective PRMT5 inhibitor that has been shown to exert antiproliferative and antitumor activity in several hematological and solid malignancies (23–27). At this point, it remains unknown whether PRMT5 plays a role in angiogenesis, which is critically involved in tumor growth, metastasis, and development of peripheral vascular diseases.

In this study, we sought to investigate the role of PRMT5 in hypoxia-induced angiogenesis and molecular mechanisms involved. We showed that genetic and pharmacological inhibition of PRMT5 markedly attenuated hindlimb ischemia–induced angiogenesis and VEGF-induced endothelial proliferation, migration, and tube formation, through decreasing HIF-1α protein stability and suppressing VEGF-induced signal transduction in vascular endothelial cells (ECs).

## Results

### PRMT5 is upregulated under hypoxia.

**Figure 1:**
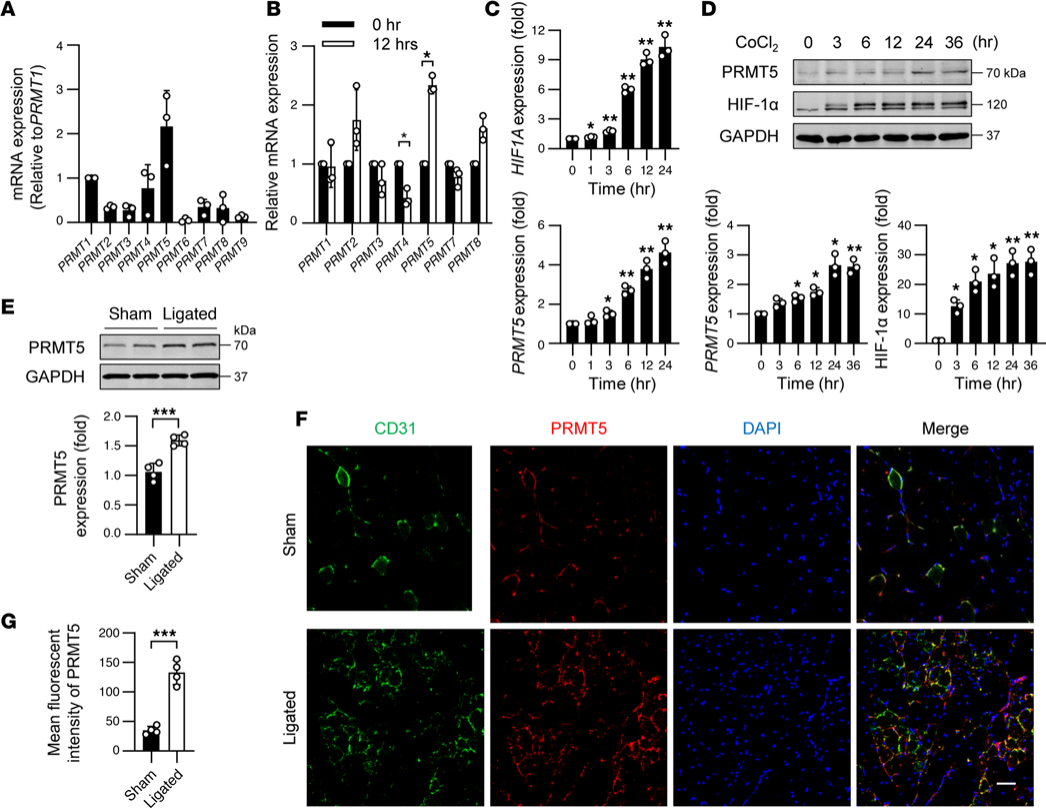
PRMT5 was upregulated under hypoxia. (**A**) Relative expression of *PRMT1–9* in HUVECs was determined by qPCR. *n* = 3. (**B**) HUVECs were treated with 200 μM CoCl_2_ for 0 and 12 hours, and the mRNA levels of *PRMT1–8* were determined by qPCR. *P < 0.05 by 2-tailed Student’s *t* test. *n* = 3. (**C**) HUVECs were treated with 200 μM CoCl_2_ for indicated time periods, and the RNA levels of *HIF1A* and *PRMT5* were determined by qPCR. *n* = 3. (**D**) HUVECs were treated with 200 μM CoCl_2_ for indicated time periods in complete ECM. The expression of PRMT5, HIF-1α, and GAPDH was determined by Western blot and then quantitated by densitometric analysis. *n* = 3. *P < 0.05, **P < 0.01 versus 0 hours using 1-way ANOVA coupled with Tukey’s multiple-comparison post hoc test. (**E**) Western blot analysis of PRMT5 and GAPDH protein expression in GC muscles isolated from ligated and nonligated (sham) hindlimbs of WT mice. Protein levels were quantitated by densitometric analysis. ***P < 0.001 compared with sham group, using 2-tailed Student’s *t* test. *n* = 4. (**F**) Sections of GC muscles were stained with CD31 (green), PRMT5 (red), and DAPI (blue) to show expression of PRMT5 in the ligated and sham hindlimbs. The representative images were shown. Scale bars: 50 μm. *n* = 4. (**G**) Quantification of mean fluorescence intensity of PRMT5 in the ROI of CD31^+^ regions. ***P < 0.001, using 2-tailed Student’s *t* test, *n* = 4. Data are shown as mean ± SD.

To define the role of PRMTs in angiogenesis, we first determined the relative expression of *PRMTs* in human umbilical vein ECs (HUVECs), which express many important endothelial markers and signaling molecules and are widely used for in vitro studies of the vasculature and angiogenesis (28). Among the 9 members of *PRMT*, we found that *PRMT5* was highly expressed in ECs (Figure 1A), and its expression was significantly increased in response to the treatment of cobalt chloride (CoCl_2_) (Figure 1B), which has been shown to simulate hypoxia in various in vitro studies (29), while the expression of *PRMT6* and *PRMT9* was barely detected under both basal and hypoxic conditions. Similar to the expression of HIF-1α, we found that, in a time-dependent manner, both mRNA and protein levels of PRMT5 were significantly increased in response to CoCl_2_ treatment (Figure 1, C and D). Twenty-four hours after CoCl_2_ treatment, PRMT5 protein was increased by approximately 2.5-fold. To determine whether PRMT5 plays a role in hypoxia-induced angiogenesis, we examined the expression of PRMT5 in hypoxic tissues. As shown in Figure 1E, in a mouse model of hindlimb ischemia (30), the expression of PRMT5 in the ligated side in gastrocnemius (GC) muscles was significantly higher than that in the nonligated side. Increased expression of PRMT5 in the ligated side was primarily in ECs, as determined by immunofluorescent staining of PRMT5 and CD31 (Figure 1, F and G). These results suggest that PRMT5 expression was increased in the ischemic hindlimbs of mice, and its localization was largely overlapped with the hypoxia-induced new capillaries, indicating a potential role of PRMT5 in ischemia-induced neovascularization.

### Generation of inducible EC–specific Prmt5-KO mice.

**Figure 2:**
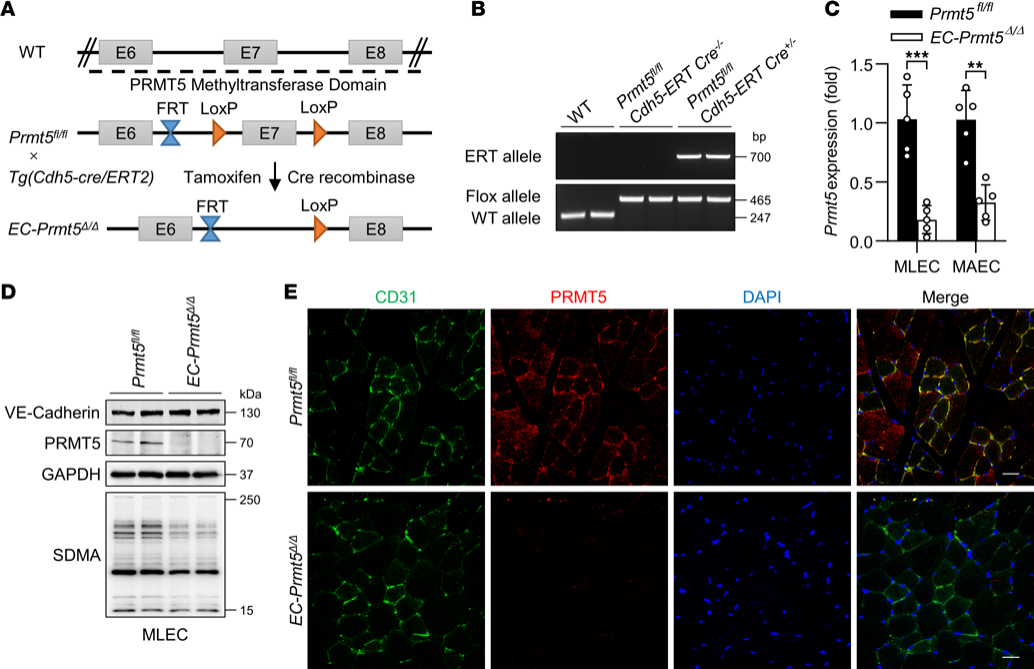
Generation of inducible EC–specific *Prmt5* KO mice. (**A**) Strategy of generating inducible endothelial cell specific *Prmt5* knockout mice. (**B**) Genotyping results of genomic RNA isolated from WT, *Prmt5^fl/fl^*/*Cdh5 ERT Cre^–/–^* and *Prmt5^fl/fl^*/*Cdh5 ERT Cre^+/–^* (*EC*-*Prmt5^Δ/Δ^*) mice. (**C**) The mRNA levels of *Prmt5* in MLECs and MAECs isolated from *Prmt5^fl/fl^* and *EC-Prmt5^Δ/Δ^* mice were determined by qPCR. **P < 0.01, ***P < 0.001, using 2-tailed Student’s *t* test. Data are representative of mean ± SD. *n* = 5. (**D**) Levels of PRMT5 protein and symmetric dimethyl arginine (SDMA) in MLECs isolated from *Prmt5^fl/fl^* and *EC-Prmt5^Δ/Δ^* mice were determined by Western blot. VE-Cadherin was shown as the endothelial marker. *n* = 4. (**E**) Immunofluorescent staining of CD31 (green), PRMT5 (red), and DAPI (blue) to show the expression of PRMT5 in blood vessels of GC muscles obtained from *Prmt5^fl/fl^* and *EC-Prmt5^Δ/Δ^* mice. Scale bars: 20 μm. *n* = 4.

PRMT5 plays an important role in normal development, and global KO of *Prmt5* has been shown to induce embryonic lethality (17). To define the role of PRMT5 in ECs, we generated inducible EC–specific *Prmt5*-KO mice by crossing *Prmt5^fl/fl^* mice with *Cdh5-ERT/Cre* transgenic mice to generate *Prmt5^fl/fl^/Cdh5-ERT/Cre^+/–^* inducible mice. To delete the *Prmt5* gene in ECs, 5 i.p. injections of tamoxifen (75 mg/kg, days 1–5) were given to both *Prmt5^fl/fl^/Cdh5-ERT/Cre^+/–^* and *Prmt5^fl/fl^* mice. After recombination of LoxP, the exon 7 that encodes part of PRMT5 methyltransferase domain was removed in *EC*-*Prmt5*^Δ*/*Δ^ mice (Figure 2A). Mouse genotype was confirmed by PCR analysis of genomic DNA obtained from mice tails (Figure 2B). To further verify the KO efficacy of *Prmt5* in ECs, murine lung ECs (MLECs) and murine aortic ECs (MAECs) from *Prmt5^fl/fl^* and *EC*-*Prmt5*^Δ*/*Δ^ mice were isolated to detect the mRNA levels of *Prmt5* using quantitative PCR (qPCR). As shown in Figure 2C, the mRNA levels of *Prmt5* in MLECs and MAECs isolated from *EC*-*Prmt5*^Δ*/*Δ^ mice were significantly decreased compared with the *Prmt5^fl/fl^* group. In addition, Western blot demonstrated similar results as shown in Figure 2D. The SDMA products mainly catalyzed by PRMT5 were significantly decreased in *Prmt5*-depleted ECs. Immunofluorescent staining indicated that PRMT5 expression was virtually absent in mouse blood vessels as indicated by immunofluorescent staining of PRMT5 and CD31 in the blood vessels of GC muscles of *Prmt5^fl/fl^* and *EC*-*Prmt5*^Δ*/*Δ^ mice (Figure 2E), further indicating a successful deletion of *Prmt5* in vascular ECs after tamoxifen injections.

### EC deletion of Prmt5 impairs angiogenesis after hindlimb ischemia.

**Figure 3:**
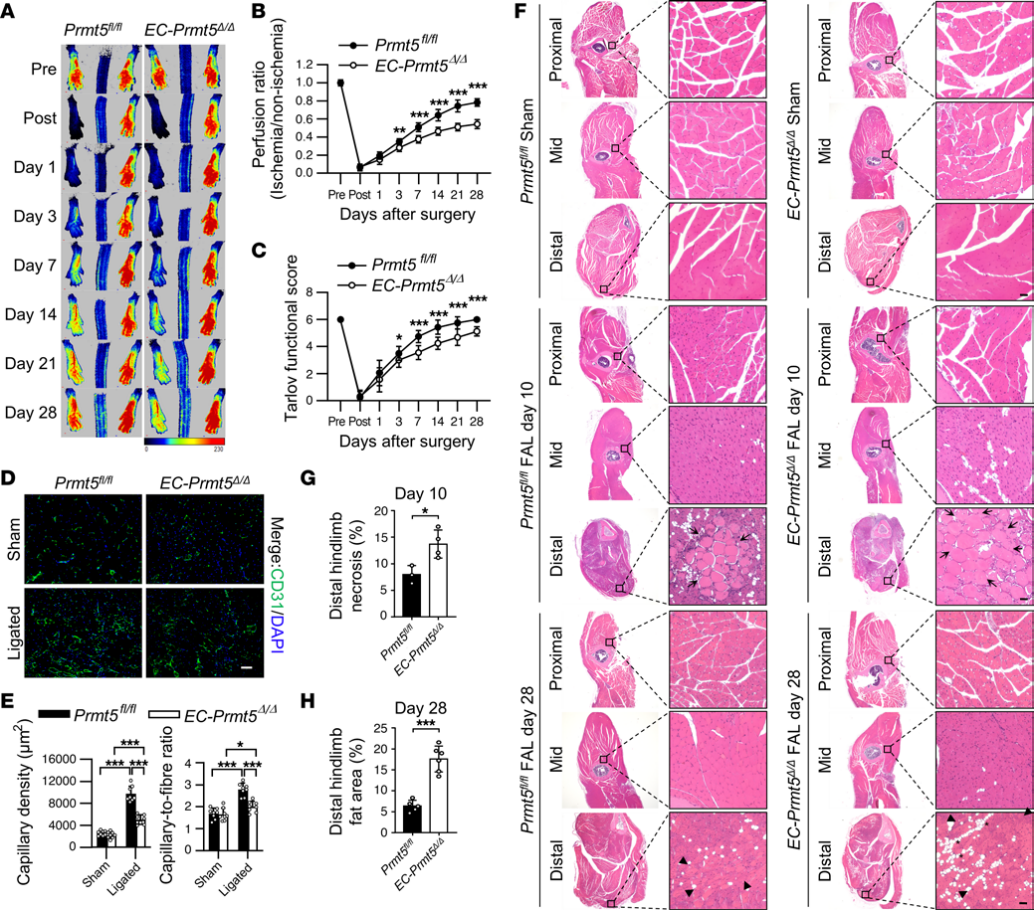
Impairment of blood flow recovery and angiogenesis after hindlimb ischemia induced by femoral artery ligation (FAL) in EC-specific *Prmt5*-KO mice. (**A**) Laser Speckle Contrast Imaging (LSCI) was used to measure the blood perfusion in hindlimb of mice, and blood flow was shown on a blue/black-to-red scale, with red indicating greater perfusion. *n* = 8. (**B**) The blood perfusion monitoring in the ischemic hindlimbs was quantified using the nonischemic hindlimbs as reference. **P < 0.01 and ***P < 0.001 versus *Prmt5^fl/fl^* group at the same time point, using 2-tailed Student’s *t* test. *n* = 8. (**C**) Tarlov functional score of the hindlimbs in both groups. *P < 0.05 and ***P < 0.001 versus *Prmt5^fl/fl^* group at same time point, using Mann-Whitney *U* test. *n* = 8. (**D**) Immunofluorescent staining of CD31 (green) and DAPI (blue) in GC muscles of the ischemic and nonischemic (sham) hindlimbs. Scale bar: 50 μm. *n* = 8. (**E**) Quantitation of capillaries area and ratio of capillary/muscle fibers. *P < 0.05 and ***P < 0.001, using 2-way ANOVA coupled with Tukey’s multiple-comparison post hoc test. *n* = 8. (**F**) H&E-stained full cross-section of the sham and ligated hindlimbs, harvested at 10 days or 28 days after surgery. Injury induced necrosis (arrows) at day 10, regeneration (arrow heads), and adipocyte deposition (asterisks) at day 28 were shown in GC muscle of distal hind limb. Scale bars: 30 μm. *n* = 8–10. (**G**) Quantitation of injury-induced necrosis in distal ischemic hindlimbs from *Prmt5^fl/fl^* and *EC-Prmt5^Δ/Δ^* mice after 10 days of FAL. *P < 0.05, using 2-tailed Student’s *t* test. *n* = 4. (**H**) Quantitation of the fat area as shown by adipocytes deposition in GC muscle fibers of distal ischemic hindlimbs from *Prmt5^fl/fl^* and *EC-Prmt5^Δ/Δ^* mice at 28 days of FAL. ***P < 0.001, using 2-tailed Student’s *t* test. *n* = 5 or 6. All data were shown as mean ± SD.

To substantiate the function of PRMT5 in angiogenesis, a mouse model of hindlimb ischemia was established, and the angiogenesis was determined by measuring blood flow recovery in the hindlimb and the area of new capillaries in distal ischemic GC. *Prmt5^fl/fl^* and *EC*-*Prmt5*^Δ*/*Δ^ mice were subjected to the femoral artery ligation, and the blood flow in the ischemic and nonischemic limbs was compared at different time points after ligation using Laser Speckle Contrast Imaging (LSCI) (Figure 3A). The blood flow ratios in both groups were 1.0 before surgery but decreased to nearly 5% after surgery, indicating that the hindlimb ischemia model was successfully established. The blood flow in ischemic hindlimbs gradually recovered in both groups. However, the recovery was significantly impaired in *EC*-*Prmt5*^Δ*/*Δ^ mice at 3, 7, 14, 21, and 28 days after ligation, as compared with the control mice (Figure 3B). Similarly, the femoral artery ligation of hindlimb caused ischemia in the lower extremity, making the mice almost incapacitated in both groups. As time went by, the hindlimb movement gradually recovered; however, *EC*-*Prmt5*^Δ*/*Δ^ mice recovered more slowly than *Prmt5^fl/fl^* mice at 3, 7, 14, 21, and 28 days after surgery (Figure 3C). Ligation of the femoral artery in the hindlimb led to ischemia and hypoxia in the distal GC muscle, thus inducing angiogenesis (30). The GC muscles were collected and stained with CD31 to assess the new capillaries. Compared with nonligated hindlimbs, the capillary density and capillary/fiber ratio of GC muscles were significantly increased in ligated hindlimbs in both groups. However, the capillary area in the *EC*-*Prmt5*^Δ*/*Δ^ group was significantly decreased compared with the control group (Figure 3, D and E). Inadequate angiogenesis in ligated hindlimbs often leads to necrosis in GC muscles, as characterized by multicellular infiltration and the appearance of hypereosinophilic muscle cells devoid of nuclei (30, 31). To examine the muscle injury and regeneration after hindlimb ischemia, whole hindlimbs were collected at 10 and 28 days after ligation injury for the H&E staining. As shown in Figure 3F, the muscle fibers in nonligated sham hindlimbs were neatly aligned in both groups. At day 10 after ligation, injury-induced muscle necrosis — particularly in GC muscle of distal hindlimbs, as characterized by disrupted myofibers devoid of nuclei — was exacerbated in *EC*-*Prmt5*^Δ*/*Δ^ mice as compared with their WT littermates (Figure 3, F and G). Twenty-eight days after the ligation, angiogenesis and capillary regeneration were stabilized, and injury-induced regenerated areas, as characterized by pale myofibers with centralized nuclei, were observed in ischemic GC muscles of both groups. However, compared with the WT group, the accumulation of adipocytes among regenerated GC muscle fibers, which indicates impaired muscle recovery (30), was substantially increased in the *EC*-*Prmt5*^Δ*/*Δ^ group, suggesting a decreased muscle degeneration in these mice (Figure 3, F and H). Furthermore, endothelial *Prmt5* KO led to a decreased arteriogenesis induced by ligation, as indicated by a reduction in femoral collateral artery wall thickening in semimembranosus muscles (Supplemental Figure 1; supplemental material available online with this article; https://doi.org/10.1172/jci.insight.152481DS1). Together, these results suggest that *Prmt5* KO in ECs attenuates ischemia-induced angiogenesis and injury-induced muscle regeneration.

### Inhibition of PRMT5 attenuates aortic ring sprouting and tube formation on Matrigel.

**Figure 4:**
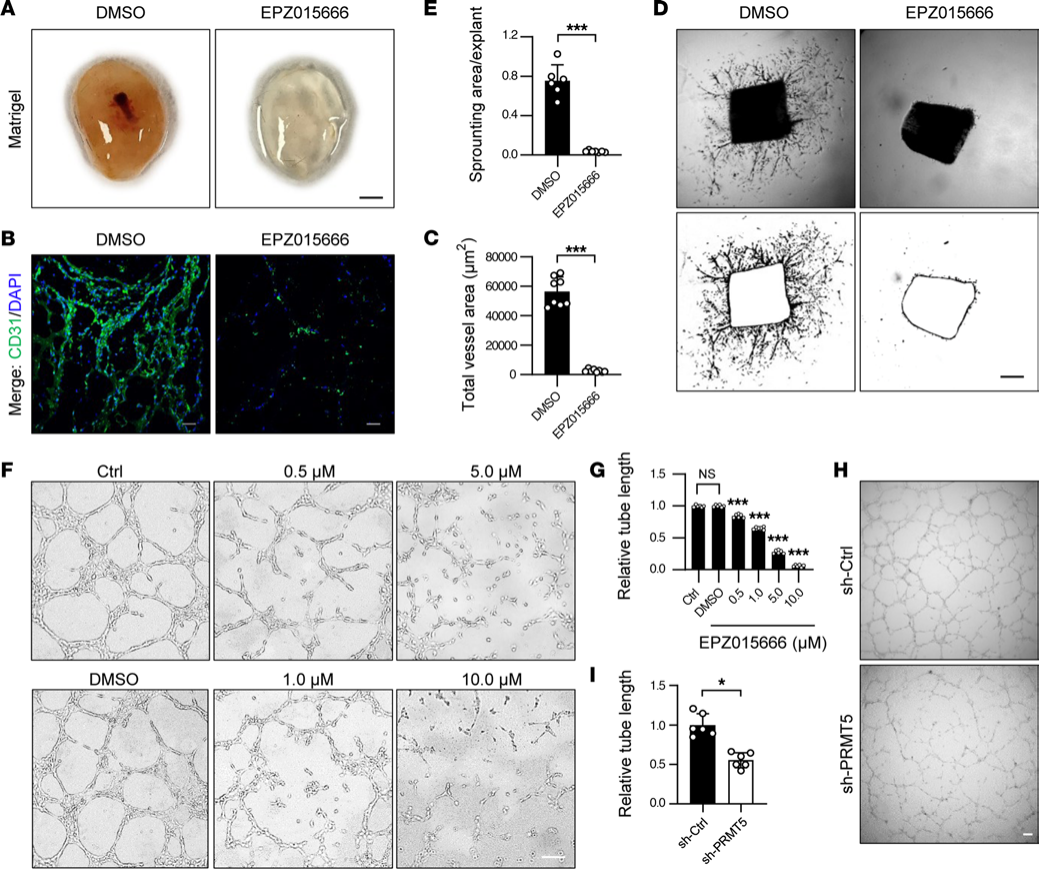
PRMT5 inhibitor attenuates aortic ring sprouting and tube formation on Matrigel. (**A**) PRMT5 inhibitor EPZ015666 attenuates neovascularization in the Matrigel plug assays. The VEGF-containing Matrigel plugs with or without 10.0 μM EPZ015666 (dissolved in DMSO, final concentration 0.1%) were implanted and harvested from WT mice after 14 days. The Matrigel plugs were washed and photographed. Scale bar: 2.5 mm. *n* = 8. (**B** and **C**) Sections of Matrigel plugs were double stained with CD31 (green) and DAPI (blue). The representative image and quantification of total vessel areas in each Matrigel plug are shown. Scale bars: 50 μm. ***P < 0.001, using 2-tailed Student’s *t* test. *n* = 8. (**D** and **E**) The thoracic aortic ring isolated from WT mice were cultured in VEGF-containing Matrigel with or without 10.0 μM EPZ015666 for 4–5 days. The new vessels sprouting was captured and represented as an outline. The representative images and quantification are shown. Scale bar: 200 μm. ***P < 0.001, using 2-tailed Student’s *t* test. *n* = 6. (**F** and **G**) HUVECs were incubated with 0.1% DMSO or 0.5, 1.0, 5.0, or 10.0 μM EPZ015666 for 4 days and then seeded on Matrigel combined with different concentrations of EPZ015666. Eight hours after seeding, tube formation was captured using EVOS imaging system. The representative images and quantification were shown. Scale bar: 100 μm. ***P < 0.001 versus DMSO, using 1-way ANOVA coupled with Tukey’s multiple-comparison post hoc test. *n* = 6. (**H** and **I**) HUVECs were infected with lentivirus expressing control shRNA (sh-Ctrl) or PRMT5 shRNA (sh-PRMT5). Seventy-two hours after infection, cells were harvested and then seeded on Matrigel for an additional 8 hours to detect tube formation. The representative images and quantification were shown. Scale bar: 100 μm. *P < 0.05, using 2-tailed Student’s *t* test. *n* = 6. All data were shown as mean ± SD.

Neovascularization, vessel sprouting, and EC tube formation have been shown to contribute significantly to blood flow recovery in ischemic hindlimbs (32). Therefore, we performed Matrigel plug, aortic ring sprouting, and tube formation assays to examine the effects of pharmacological inhibition of PRMT5 on angiogenesis. EPZ015666 (GSK3235025) is a potent PRMT5 inhibitor (Supplemental Figure 2A) that is under clinical investigation for cancer treatment (23). In ECs, we showed that EPZ015666 inhibited PRMT5 activity in a time- and dose-dependent manner, as shown by reduced production of its methylation product SDMA (Supplemental Figure 2, B and C). Thus, we performed a Matrigel plug assay to analyze the effect of EPZ015666 on angiogenesis. As shown in Figure 4A, the Matrigel plug containing PRMT5 inhibitor EPZ015666 was mostly colorless and transparent, while in the DMSO control group, small blood vessels with hemoglobin were clearly observed. In VEGF-containing Matrigel plugs, the total newly formed vessel area was significantly lower in the EPZ015666 group than that in DMSO control group, as determined by CD31 immunofluorescent staining (Figure 4, B and C). Furthermore, inhibition of PRMT5 by EPZ015666 significantly attenuated VEGF-induced aortic ring sprouting (Figure 4, D and E), as well as tube formation, in a dose-dependent manner (Figure 4, F and G). Likewise, lentiviral shRNA–mediated knockdown of *PRMT5* also significantly attenuated the tube formation of ECs (Figure 4, H and I). Together, these data suggest that PRMT5 plays a crucial role in VEGF-induced ex vivo neovascularization, aortic ring sprouting, and EC tube formation on Matrigel.

### Inhibition of PRMT5 attenuates proliferation and migration of ECs.

**Figure 5:**
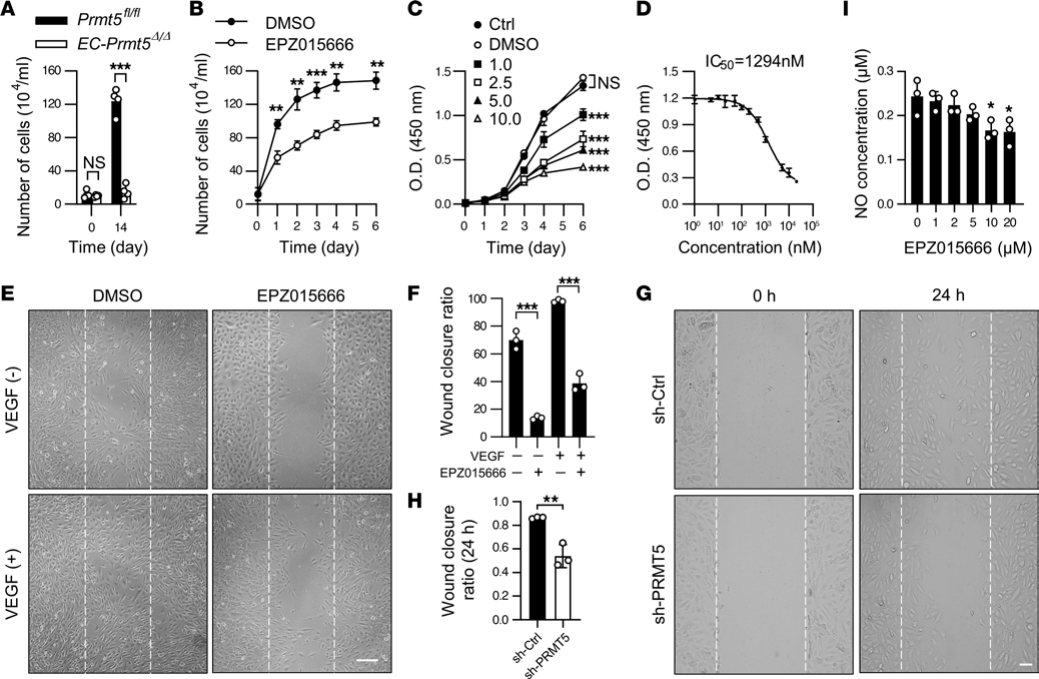
Inhibition of PRMT5 attenuates endothelial cell proliferation and migration. (**A**) MLECs isolated from *Prmt5^fl/fl^* and *EC-Prmt5^Δ/Δ^* mice were cultured in complete ECM and were counted at days 0 and 14. ***P < 0.001, using 2-tailed Student’s *t* test. *n* = 4. (**B**) HUVECs were incubated with 0.1% DMSO or 10.0 μM EPZ015666 for indicated days, and the cell numbers were calculated by hemocytometer. **P < 0.01 and ***P < 0.001 versus 0.1% DMSO at same time point, using 2-tailed Student’s *t* test. *n* = 3. (**C**) HUVECs were seeded into a 96-well plate at 5%–10% density and incubated with either 0.1% DMSO or EPZ015666 at indicated doses for 0, 1, 2, 3, 4, and 6 days. Cell proliferation was determined by CCK-8 assay. ***P < 0.001 versus 0.1% DMSO, using 2-way ANOVA coupled with Tukey’s multiple-comparison post hoc test. *n* = 3. (**D**) CCK-8 assay was performed in HUVECs incubated with increasing concentrations of PRMT5 inhibitor EPZ015666 for 6 days. *n* =3, IC_50_ = 1294 nM. (**E** and **F**) Wound healing assay was performed in HUVECs incubated with 0.1% DMSO or 10.0 μM EPZ015666 for 4 days, in the presence or absence of VEGF stimulation (50 ng/mL). The representative images and quantification are shown. Scale bars: 100 μm. ***P < 0.001, using 2-tailed Student’s *t* test. *n* = 3. (**G** and **H**) HUVECs were infected with lentivirus expressing control shRNA (sh-Ctrl) or PRMT5 shRNA (sh-PRMT5). Three days after infection, wound healing assays were performed to determine cell migration. The representative images and quantification are shown. Scale bar: 100 μm. **P < 0.01, using 2-tailed Student’s *t* test. *n* = 3. (**I**) HUVECs were treated with EPZ015666 at indicated doses for 3 days, followed by 12-hour starvation. NO concentration in the culture media was determined after stimulation of VEGF for 6 hours. *P < 0.05 versus 0 μM, using 1-way ANOVA coupled with Tukey’s post hoc test. *n* = 3. All data were exhibited as mean ± SD.

EC proliferation and migration play essential roles in neovascularization. We then isolated MLECs from *Prmt5^fl/fl^* and *EC*-*Prmt5*^Δ*/*Δ^ mice, and we examined their proliferation rates in the presence of EC complete culture medium. As shown in Figure 5A, the initial number of cells after isolation from lungs was not different between the 2 groups. However, after 14 day of culture, the number of MLECs isolated from *EC*-*Prmt5*^Δ*/*Δ^ was significantly less than that from *Prmt5^fl/fl^* mice. Similarly, pharmacological inhibition of PRMT5 by EPZ015666 markedly inhibited HUVEC proliferation in a dose-dependent manner, with an IC_50_ of 1.3 μM, as determined by CCK-8 assays (Figure 5, B–D). Furthermore, we found that both EPZ015666 and lentiviral shRNA–mediated reduced activity of PRMT5 significantly inhibited VEGF-induced EC migration (Figure 5, E–H). Nitric oxide (NO) is essential for maintaining EC proliferation and vascular homeostasis (33). To determine whether PRMT5 impacts NO production, we measured NO production in EPZ015666-treated ECs after VEGF-A stimulation for 6 hours. As shown in Figure 5I, EPZ015666 treatment reduced NO synthesis in a dose-dependent manner. Together, these results indicate that inhibition of PRMT5 attenuates in vitro EC proliferation and migration.

### Inhibition of PRMT5 attenuates VEGF/PI3K/eNOS signaling pathway.

**Figure 6:**
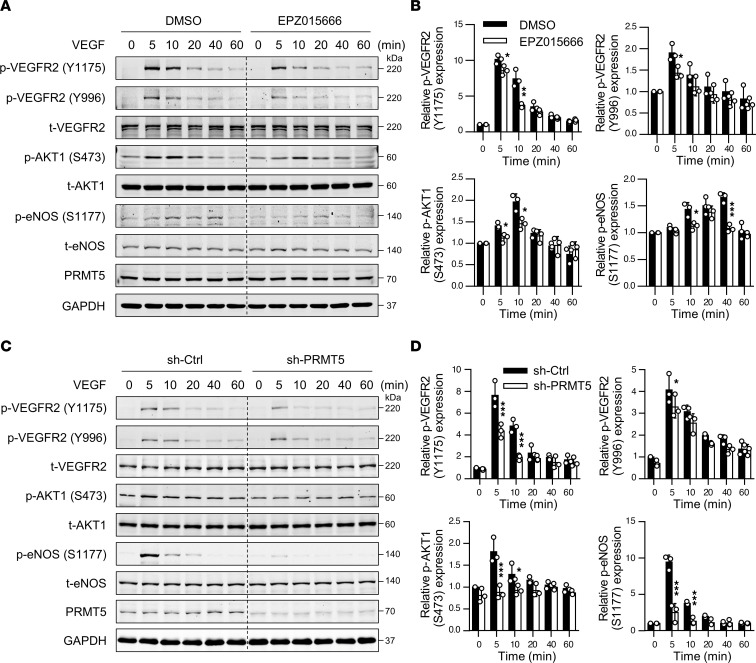
Inhibition of PRMT5 attenuates the VEGF/VEGFR2/PI3K/eNOS signaling pathway. (**A**) HUVECs at around 70% density were incubated with 0.1% DMSO or 10.0 μM EPZ015666 for 4 days. After starvation, cells were treated with VEGF (50 ng/mL) and harvested at indicated time points. Western blot was performed to determine the levels of p-VEGFR2 (Y996), p-VEGFR2 (Y1175), t-VEGFR2, p-eNOS (S1177), t-eNOS, p-AKT1 (S-473), t-AKT1, PRMT5, and GAPDH protein. *n* = 3. (**B**) Quantification of phosphorylated protein expression by densitometric analysis. *P < 0.05, **P < 0.01, and ***P < 0.001 versus DMSO group at the same time point, using 2-tailed Student’s *t* test. *n* = 3. (**C**) HUVECs were incubated with sh-Ctrl or sh-PRMT5 for 3 days, followed by starvation and VEGF stimulation to detect phosphorylation of VEGFR2, eNOS, and AKT1. *n* = 3. (**D**) Quantification of phosphorylated proteins by densitometric analysis. *P < 0.05 and ***P < 0.001 versus sh-Ctrl group at the same time point, using 2-tailed Student’s *t* test. *n* = 3. All data were exhibited as mean ± SD.

VEGF-A is one of the most prominent angiogenetic factors implicated in angiogenesis (34). Signal transduction pathways initiated by VEGF-A/VEGFR2 lead to EC proliferation, migration, survival, and abnormal angiogenesis (35). To elucidate the molecular mechanism involved in regulating angiogenesis by PRMT5, we investigated whether PRMT5 inhibitor EPZ015666 impacts VEGF/VEGFR-mediated signaling pathways in ECs. To this end, HUVECs were pretreated with 10.0 μM EPZ015666 for 4 days and then treated with 50 ng/mL VEGF-A for 0, 5, 10, 20, 40, and 60 minutes. Phosphorylation of VEGF downstream signaling molecules was then determined by Western blot. As shown in Figure 6, both inhibition of PRMT5 by EPZ015666 and lentiviral shRNA–mediated knockdown markedly inhibited VEGF-A–induced VEGFR2 phosphorylation at Y996 and Y1175, while the total expression of VEGFR2 was barely affected. In addition, both EPZ015666 treatment and PRMT5 knockdown attenuated VEGF-A–induced phosphorylation of AKT1 at S-473 and endothelial NO synthase (eNOS) at S-1177, both of which are critically involved in promoting EC proliferation, migration, and angiogenesis (35). These findings indicate that PRMT5 is essentially involved in VEGF-A–induced VEGFR2 signaling pathways in ECs.

### Inhibition of PRMT5 decreases the expression and stability of HIF-1α–induced by hypoxia.

**Figure 7:**
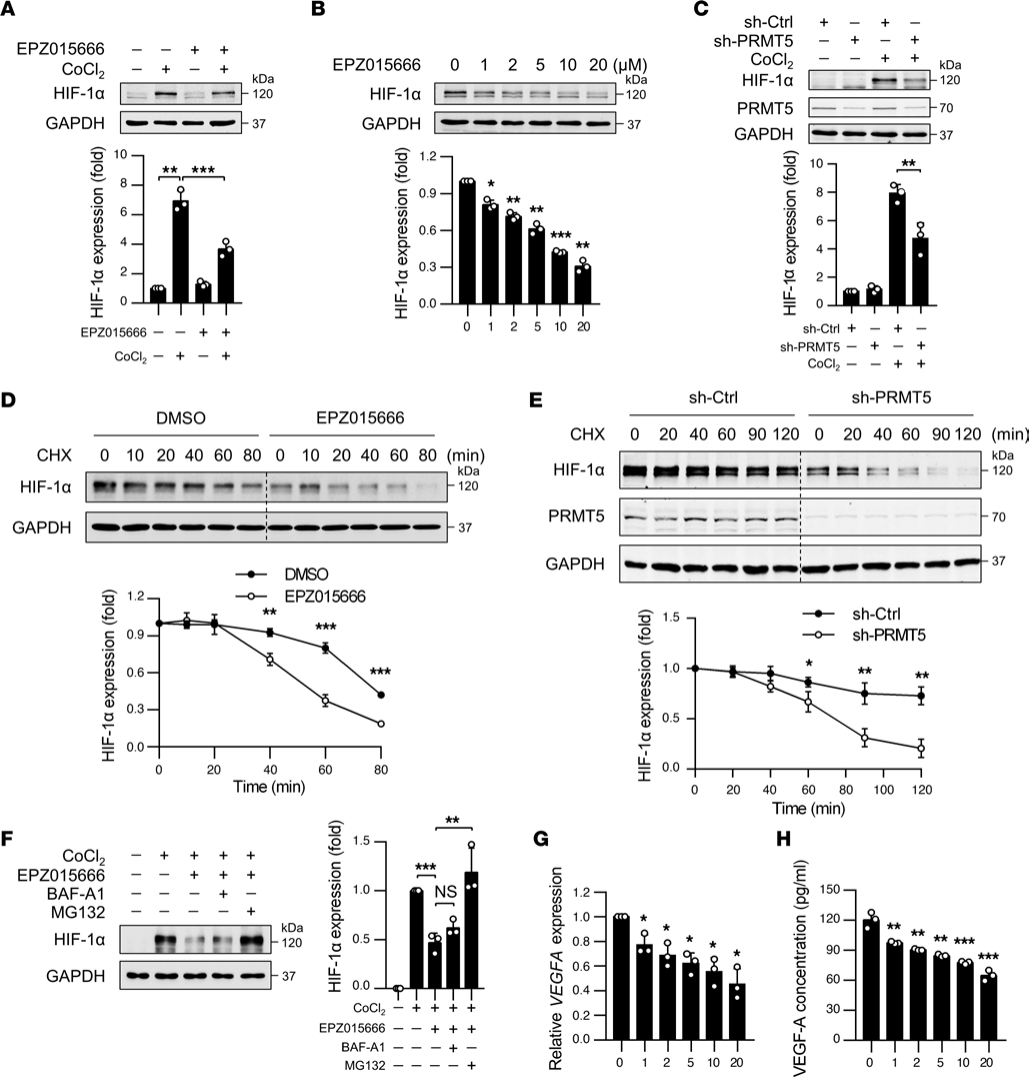
PRMT5 inhibitor decreased the expression and stability of HIF-1α induced by hypoxia. (**A**) HUVECs were incubated with either vehicle or 10.0 μM EPZ015666 for 4 days before exposure to 200 μM CoCl_2_ for 24 hours. The expression of HIF-1α was determined by Western blot. *n* = 3. (**B**) Dose-dependent effects of EPZ015666 on the expression of HIF-1α in HUVECs as determined by Western blot. *n* = 3. (**C**) HUVECs were incubated with lentivirus expressing sh-Ctrl or sh-PRMT5 for 72 hours, followed by CoCl_2_ treatment. Expression of indicated proteins was determined by Western blot. *n* = 3. (**D**) The stability of HIF-1α in EPZ015666-treated cells was determined by Western blot in the presence of 20 μg/mL cycloheximide (CHX). **P < 0.01, ***P < 0.001 versus 0.1% DMSO at same time point, using 2-tailed Student’s *t* test. *n* = 3. (**E**) The stability of HIF-1α in PRMT5 knockdown cells was determined by Western blot. *P < 0.05 and **P < 0.01 versus sh-Ctrl at same time point, using 2-tailed Student’s *t* test. *n* = 3. (**F**) HUVECs were incubated with or without 10.0 μM EPZ015666 and then pretreated with MG132 (10.0 μM) or BAF-A1 (100 nM) for 30 minutes before exposure to CoCl_2_ treatment. The expression of HIF-1α was determined by Western blot. *n* = 3. (**G**) qPCR detection of *VEGFA* mRNA in ECs treated with EPZ015666. *n* = 3. (**H**) ELISA measurements of VEGF-A levels in the culture supernatants harvested from HUVECs treated with indicated concentrations of EPZ015666. *n* = 3. (**A**–**C** and **F**–**H**) *P < 0.05, **P < 0.01, ***P < 0.001, using 1-way ANOVA coupled with Tukey’s multiple-comparison post hoc test. Groups in **B**, **G**, and **H** were compared with 0 μM group. All data were exhibited as mean ± SD.

HIF-1α and its downstream proangiogenic molecules such as VEGF-A play important roles in ischemia/hypoxia-induced angiogenesis (5). Thus, we investigated whether inhibition of PRMT5 affects HIF-1α protein levels in ECs under hypoxic conditions. In a CoCl_2_-simulated hypoxic environment, the expression of HIF-1α in HUVECs was increased by approximately 7-fold, as compared with a normoxic environment. However, both inhibition of PRMT5 by EPZ015666 and knockdown of PRMT5 by lentiviral shRNA markedly attenuated CoCl_2_-induced expression of HIF-1α protein (Figure 7, A–C), while the mRNA levels of *HIF1A* was barely affected (data not shown), suggesting that a posttranslational mechanism may be involved in regulating HIF-1α protein levels by PRMT5.

To further investigate the mechanism underlying the regulation of HIF-1α by PRMT5, we determined HIF-1α protein stability in the presence of cycloheximide (CHX), which blocks translation and neo protein synthesis. In this regard, ECs were subjected to the treatment of either 10.0 μM EPZ015666 or vehicle for 2 days and then exposed to chemical hypoxia for 24 hours. Fresh complete ECM was then replaced with CHX supplement. The degradation rate of HIF-1α protein was then determined by Western blot. As shown in Figure 7D, PRMT5 inhibitor EPZ015666 decreased the stability of HIF-1α protein induced by hypoxia and accelerated its degradation. A similar result was obtained in shRNA-mediated *PRMT5*-knockdown cells (Figure 7E). Furthermore, we found that EPZ015666-induced HIF-1α degradation was prevented by the proteasome inhibitor MG132 but not by the lysosome inhibitor bafilomycin A1 (BAF-A1) (Figure 7F), suggesting that PRMT5 regulates the stability of HIF-1α through the proteasome pathway. Furthermore, the expression and production of VEGF-A, a well-characterized downstream target of HIF-1α (36), were dose-dependently inhibited by EPZ015666 (Figure 7, G and H), as determined by both qPCR and ELISA. Taken together, these results provide an additional mechanism indicating that inhibition of PRMT5 attenuated angiogenesis, at least in part, through decreasing the protein stability of HIF-1α and VEGF-A production.

## Discussion

It has been increasingly recognized that PRMTs family-catalyzed methylation of histone and nonhistone proteins play essential roles in regulating a variety of cellular processes, including chromatin regulation, cell signal transduction, RNA processing, and gene expression (12, 13). As a major type II methyltransferase, PRMT5 is widely expressed in embryonic and adult tissues, but it is expressed at a much higher level in embryonic tissues and plays an important role in normal development (37). Mice with homozygous *Prmt5* KO were unable to produce embryos (17). In addition, PRMT5 is essentially involved in the proliferation of human pluripotent stem cells, differentiation of muscle and nerve cells, primordial germ cells, and keratinocytes (37–41). However, the role of PRMT5 in cardiovascular biology remains largely unknown.

Recently, the role of PRMT5 in cancer development has received significant attention (18). Increased expression of PRMT5 is closely related to the occurrence and progression of a variety of tumors. Increasingly evidence suggests that regulation of abnormal angiogenesis by PRMT5 may contribute significantly to tumor growth and metastasis (18, 19). Studies have shown that, in mouse melanoma, PRMT5 overexpression promotes tumor growth, while deprivation of *Prmt5* inhibits tumor growth (42). Inhibition of PRMT5 can increase the abundance of infiltrating immune cells and enhance antitumor immunity by inhibiting the expression of NLRC5 and methylation of IFN inducible protein 116/204 (IFI116/IFI204) (43). In addition, the high expression of PRMT5 in lung cancer is associated with poor prognosis (20). Inhibition of PRMT5 can inhibit the phosphorylation of protein kinase AKT1, thereby affecting the growth cycle of lung cancer cells (44). On the other hand, PRMT5 has been shown to activate the AKT1 and ERK signaling pathways by modifying histone H4R3, thereby promoting metastasis of lung cancer cells (45).

While the significance of PRMT5 in tumors has been increasingly recognized (18), its roles in the cardiovascular system remain largely unclear. In cardiomyocytes, we show that PRMT5 inhibits cardiomyocyte hypertrophy through an inhibitory interaction with the transcriptional factor GATA4 (46). In ECs, PRMT5 has been shown to be essentially involved in vascular inflammation (47). PRMT5 is required for TNF-α–induced expression of CXCL10 and CXCL11 through arginine methylation of NF-κB p65 (48). Furthermore, PRMT5-mediated HOXA9 methylation is essentially involved in the expression of leukocyte adhesion molecules in ECs (49). In zebrafish studies, PRMT5 promotes vascular morphogenesis by affecting the transcriptional regulation of endothelial adhesion proteins (50). To further investigate the role of PRMT5 in EC biology, we attempted to generate EC-specific *Prmt5*-KO mice; however, we found that the constitutive deletion of *Prmt5* in ECs led to embryonic lethality, suggesting that PRMT5 is essential for appropriate vascular remodeling during development. Thus, we created inducible EC–specific *Prmt5-*KO mice to study the endothelial role of PRMT5 in adult mice. After tamoxifen injection, the expression of PRMT5 was substantially decreased in ECs. Furthermore, we found that endothelial-specific deletion of *Prmt5* substantially attenuated limb ischemia–induced neovascularization and EC proliferation. While the detailed molecular mechanism underlying this process is still under investigation, our results suggest that PRMT5 essentially regulates angiogenesis, at least in part, through 2 distinct pathways, including HIF-1α–mediated VEGF production and VEGFR2-mediated signaling pathway in ECs. It should be noted that Cdh5-cre–dependent recombination may also induce deletion of target genes in hematopoietic cells, and this could be a potential confounder in experiments attempting to assess the endothelial specific effects (51). Future studies performing a preceding BM transplantation will help to further address the endothelial-specific role of PRMT5 in angiogenesis.

Abnormal angiogenesis is necessary for the growth, survival, and metastasis of most solid tumors, and it involves the coordinated roles of many bioactive molecules (3). For instance, an imbalance between proangiogenic factors, such as the VEGF family, and angiogenic inhibitors, such as endostatin and angiostatin, results in abnormal angiogenesis (52). The VEGF family proteins, which include VEGF-A, VEGF-B, VEGF-C, and VEGF-D, are the best-characterized regulators implicated in many human diseases associated with angiogenesis (34). Interaction of VEGF-A with its receptor VEGFR2 results in the activation of multiple downstream targets, such as PI3K/AKT1 and eNOS, leading to increased EC proliferation, migration, and angiogenesis (35). Identification of essential regulators in this process will lead to development of novel therapies for cancer, diabetic retinopathy, wound healing, and ischemic heart disease (52). In the present study, we show that inhibition of PRMT5 markedly attenuated VEGF-A–induced phosphorylation of the VEGFR2/PI3K/AKT1/eNOS pathway in ECs. Indeed, PRMT5 has been previously shown to regulate the signal pathways of several growth factor receptors by direct arginine methylation and subsequent protein degradation of the receptors (53, 54). In our study, we found that the total protein levels of VEGFR2, AKT1, and eNOS were barely affected by PRMT5 inhibitor (Figure 6), suggesting that an indirect mechanism may be involved in regulating VEGF/VEGFR2 signal pathways by PRMT5. Nevertheless, the molecular mechanisms involved in this process warrant further investigation.

Hypoxia is associated with changes in protein arginine methylation. The expression of methionine adenosyltransferase and the formation of S-adenosyl-1-methionine are decreased under hypoxic conditions (55). In addition, PRMT2 expression and asymmetric dimethylarginine (ADMA) levels were significantly increased in chronically hypoxic mice (56). HIF-1 is a primary transcriptional factor in hypoxic response, and its expression is transcriptionally inhibited by PRMT1 (57). In this study, we demonstrate that PRMT5 is upregulated in ischemic tissues and hypoxic ECs, implicating an essential role of PRMT5 in hypoxia responses in ECs. Importantly, we found that PRMT5 is required for maintaining HIF-1α protein stability in ECs under hypoxic conditions. The detailed mechanism underlying the regulation of HIF-1α protein stability by PRMT5 is under active investigation.

In summary, we identified PRMT5 as a critical regulator in hypoxia-induced angiogenesis, due, at least in part, to its actions on HIF-1α and VEGF/VEGFR2/AKT1/eNOS pathways. Since several PRMT5 inhibitors are under preclinical and clinical studies for the treatment of cancer (58), our results suggest that PRMT5 inhibitors may have other therapeutic implications in human diseases, such as diabetic retinopathy, macular degeneration, and diabetic nephropathy. Meanwhile, these inhibitors have potential side effects, such as heart attack, hypertension, and impaired wound healing, and these effects need careful evaluation.

## Methods

### Mice and genotyping.

*Prmt5^fl/fl^* mice were provided by Stephen D. Nimer at the University of Miami (Miami, Florida, USA) (59), and C57BL/6J *Tg(Cdh5-cre/ERT2)1Rha* mice were obtained from Ralf H. Adams (The Max Planck Institute for Molecular Biomedicine, Göttingen, Germany) (60). *Prmt5^fl/fl^* mice were bred with *Tg(Cdh5-cre/ERT2)1Rha* mice to create inducible EC–specific *Prmt5*-KO mice (*Prmt5^fl/fl^/Cdh5-ERT/Cre^+/–^*). *Prmt5* gene deletion in ECs of *Prmt5^fl/fl^/Cdh5-ERT/Cre^+/–^* mice was induced by i.p. injection of tamoxifen (MilliporeSigma) at a dose of 75 mg/kg body weight for 5 consecutive days, and mice were allowed to recover for 1 week before being used for experiments. All mice were genotyped by PCR. The genomic DNA isolated from mice tail biopsies was analyzed by PCR to detect *Prmt5* WT allele (product size 247 bp), loxP-flanked allele (465 bp), and *VE-Cadherin-Cre* allele (estrogen receptor [ERT], around 700 bp). Genotyping primers for *Prmt5* allele were 5′-TGGAACTGCAGGCATATGCC-3′ (forward [F]) and 5′-TTCTGGCCTCCATGGGGGAA-3′ (reverse [R]), and the *ERT* allele were 5′-ATCCGAAAAGAAAACGTTGA-3′ (F) and 5′-ATCCAGGTTACGGATATAGT-3′ (R). All mice were on the C57BL/6J background and were maintained under specific pathogen–free conditions at 22°C with a 12-hour light/ 12-hour dark cycle. Age-matched male littermates (8–12 weeks old) were used for the experiments.

### Cell culture and hypoxia.

HUVECs were obtained from Thermo Fisher Scientific (catalog C0035C) and cultured in complete EC medium (ECM; ScienCell) supplemented with 5% FBS (ScienCell), 1% EC growth supplement (ECGS; ScienCell), and 1% antibiotic solution (penicillin/streptomycin [P/S], ScienCell) under 5% CO_2_ at 37°C. HUVEC from passage 2 to 6 were used in experiments. To induce a hypoxic environment in ECs, cells were exposed to 200 μM CoCl_2_ (MilliporeSigma) in HUVEC culture medium for 3–36 hours as a chemical hypoxia model.

### MLEC isolation.

ECs from fresh lung tissue of mice were isolated as described previously (61). Briefly, lung lobes were carefully dissected out and washed in ice-cold DMEM containing 20% FBS (Corning). Lung tissues were minced and incubated in 15 mL prewarmed HBSS (Thermo Fisher Scientific) containing 0.2 mg/mL type I collagenase (Worthington Biochemical Corporation) for 45 minutes at 37°C with shaking. After centrifugation (at 280*g* for 5 minutes at room temperature), cells were resuspended in DMEM and incubated with anti-CD31–coated (553369, BD Biosciences) Dynal magnetic beads (Thermo Fisher Scientific) on an end-over-end rotator for 10 minutes at room temperature. The beads/cell pellets were washed 5 times using the magnetic separator (Thermo Fisher Scientific) and transferred to a 0.1% gelatin-coated (MilliporeSigma) 100 mm cell culture dish (Corning) with complete ECM medium (ScienCell) supplemented with 20% FBS, 1% ECGS, and 1% P/S. To improve MLEC purity, the primary cells that reached around 80% confluence were sorted a second time using anti-CD102–coated (553326, BD Biosciences) magnetic beads.

### RNA isolation from MAEC.

Under anesthesia, mice were dissected from the midline of the abdomen and slowly perfused with PBS containing 1000 U/mL heparin (MilliporeSigma). Under a dissecting microscope, the adipose tissue attached to the thoracic aorta was carefully removed. On the distant end, the aorta was ligated by sutures. On the proximal end, a small incision was made, and the PBS solution was carefully expelled with tweezers. TRIzol (Invitrogen) reagent was slowly injected into the aorta for about 20 seconds and was then slowly withdrawn. This procedure was repeated several times. Finally, TRIzol containing MAECs was stored at –80°C for further qPCR assay.

### qPCR.

Total RNAs were extracted from ECs by using miRNeasy Mini Kit (Qiagen) or the miRNeasy Serum/Plasma Kit (Qiagen). qPCR for messenger RNA levels was performed using the miRCURY LNATM Universal cDNA Synthesis and SYBR Green Master Mix Kit (Exqion) as described (46). The primer sequences are as follows: human *PRMT1*, 5′-CTTTGACTCCTACGCACACTT-3′ (F) and 5′-GTGCCGGTTATGAAACATGGA-3′ (R); human *PRMT2*, 5′-ACATTCCGGCAAACCATGTG-3′ (F) and 5′-GGATGACTTTATCCGTCAGGGA-3′ (R); human *PRMT3*, 5′-GTACCCTTCTCATACCCCAATGG-3′ (F) and 5′-GACGAGCAGGTTCTGACATCT-3′ (R); human *PRMT4*, 5′-TCGCCACACCCAACGATTT-3′ (F) and 5′-GTACTGCACGGCAGAAGACT-3′ (R); human *PRMT5* 5′-CTGTCTTCCATCCGCGTTTCA-3′ (F) and 5′-GCAGTAGGTCTGATCGTGTCTG-3′ (R); human *PRMT6* 5′-TACCGCCTGGGTATCCTTCG-3′ (F) and 5′-CCTGTTCCGGCAACTCTACA-3′ (R); human *PRMT7* 5′-GGTGTCACCAGCCGACTTTAC-3′ (F) and 5′-TGAGCTACTGACTTGCTTGCT-3′ (R); human *PRMT8* 5′-CCTGCTAAGCCCGTGCAAT-3′ (F) and 5′-TGGGCATAGGAGTCGAAGTAA-3′ (R); human *PRMT9* 5′-GCCTGTGAGTTATCCAAGACC-3′ (F) and 5′-ACAACTAGGGACACTCTTTCGG-3′ (R); human *HIFIA*, 5′-CACCTCTTTTGGCAAGCATCCTG-3′ (F) and 5′-TATGAGCCAGAAGAACTTTTAGGC-3′ (R); human *VEGFA*, 5′-GATGGCAGTAGCTGCGCTGATA-3′ (F) and 5′-TTGCCTTGCTGCTCTACCTCCA-3′ (R); human *GAPDH*, 5′-TGTGGGCATCAATGGATTTGG-3′ (F) and 5′-ACACCATGTATTCCGGGTCAAT-3′ (R); mouse *Prmt5*, 5′-CTGAATTGCGTCCCCGAAATA-3′ (F) and 5′-AGGTTCCTGAATGAACTCCCT-3′ (R); and mouse *Gapdh*, 5′-AGGTCGGTGTGAACGGATTTG-3′ (F) and 5′-GGGGTCGTTGATGGCAACA-3′ (R).

### Western blot.

Western blot analysis was performed as described previously (46). Brieﬂy, cell lyses was resolved by SDS-PAGE and transferred to nitrocellulose membrane, followed by blocking with 5% nonfat milk in tris-buffered saline (TBS) with 0.1% Tween 20 and then incubated with diluted primary antibody at 4°C overnight. The following antibodies were used: rabbit anti-PRMT5 (1:1000, A1520, ABclonal), mouse anti–HIF-1α (1:1000, 610958, BD Biosciences), mouse anti–VE-Cadherin (1:500, sc-9989, Santa Cruz Biotechnology Inc.), rabbit anti-SDMA (1:1000, 13222S, Cell Signaling Technology), rabbit anti–p-VEGFR2 (Y1175) (1:1000, 3770S, Cell Signaling Technology), rabbit anti–p-VEGFR2 (Y996) (1:1000, 2474S, Cell Signaling Technology), rabbit anti–t-VEGFR2 (1:1000, 9698S, Cell Signaling Technology), rabbit anti–p-Akt1 (S-473) (1:1000, 9018S, Cell Signaling Technology), mouse anti–t-Akt1 (1:1000, 2920S, Cell Signaling Technology), rabbit anti–p-eNOS (S1177) (1:1000, 9571S, Cell Signaling Technology), mouse anti–t-eNOS (1:1000, 5880S, Cell Signaling Technology), or mouse anti-GAPDH (1:5000, AC002, ABclonal). After washing, secondary IRDye 680RD donkey anti-rabbit (926-68073, LI-COR) or 800CW goat anti-mouse (926-32210, LI-COR) antibodies (0.1 μg/mL) were used to visualize bands by an Odyssey Imaging System (LI-COR). Protein stability was determined in the presence of 20 μg/mL CHX (C7698, MilliporeSigma). HIF-1α degradation was determined in the presence of the proteasome inhibitor MG132 (10 μM, S2619, Selleckchem) or the lysosome inhibitor BAF-A1 (100 nM, HY-100558, MedChemExpress).

### Mouse hindlimb ischemia.

Mice were anesthetized with isoflurane inhalation and were placed on a heating mat at 37°C. The hair in the surgical area was completely removed with depilation cream, and the left lower limb area of mice was disinfected with 70% ethanol. A skin incision about 1 cm from the knee to the inside of the thigh was made to expose the femoral artery, where the anatomical markers of the femoral nerve, artery, and venous neurovascular bundle were identified. The femoral nerve, artery, and vein neurovascular bundles were separated by pulling the connective tissue next to them. After the separation, 2 surgical knots were made to ligate the proximal femoral artery. Next, the femoral artery was separated from the femoral vein distally near the knee joint, and another surgical line was placed at its lower end to close the femoral artery by 2 surgical knots. The femoral artery was transected between the distal and proximal ends of the ligated femoral artery using ophthalmic scissors. The hindlimb incisions were sutured, and the mice were s.c. injected with 0.1 mg/kg buprenorphine to relieve pain.

### LSCI.

LSCI was used to monitor blood perfusion before and after surgery and on days 1, 3, 7, 14, 21, and 28 postoperatively in both groups of mice. The laser scanning instrument (PeriCam PSI HR; PERIMED) was fixed about 11 cm above the mice, and the background threshold was adjusted. Two hindfoot regions were selected as the region of interest (ROI), and the perfusion images were obtained and quantitated using PeriCam PSI System (PERIMED).

### Tarlov functional score.

Tarlov functional scoring was used in this project to evaluate movement of hindlimbs of mice (62). The details of Tarlov functional scoring were shown in Supplemental Table 1.

### Immunofluorescent staining.

Sections were fixed with 4% formaldehyde and permeabilized with 0.2% Triton-X 100 at room temperature. Nonspecific binding was covered by incubating with 3% BSA at room temperature for 60 minutes and then incubated with rat anti-CD31 (1:500, 553369, BD Biosciences) and rabbit anti-PRMT5 (1:100, A1520, ABclonal) antibodies at 4°C overnight. Slides were washed with PBS 3 times for 5 minutes and incubated with Alexa Fluor–conjugated secondary antibodies (Alexa Fluor 488 donkey anti-rat, 1:2000, A-21208, and 555 donkey anti-rabbit, 1:2000, A-31572, Invitrogen) at room temperature for 1 hour. After staining with DAPI (1 μg/mL, Invitrogen), slides were embedded with antifade mountant reagent (Invitrogen) and photographed under a Nikon fluorescence confocal microscope.

### Tissue preparation and H&E staining.

Mice were euthanized and perfused with PBS containing 0.2% heparin, followed by 4% paraformaldehyde through the left ventricle. Whole hindlimbs were then dissected and fixed overnight. After decalcification by 10% formic acid, the hindlimbs were cut into the proximal, mid, and distal segments as described previously (31). The segments were embedded in paraffin to generate 5 μm sections for H&E staining. Skeletal muscle injury areas were identified in the GC muscle of distal hindlimb. Muscle injury induced necrosis was defined by the hypereosinophilic muscle cells devoid of nuclei coupled with multicellular infiltrates. Muscle injury–induced regeneration was identified by the presence of pale myofibers with centralized nuclei and accumulation of adipocytes (28, 29). The semimembranosus muscle is at the center of the neurovascular bundle. Collateral arteries of mice on the ligated and nonligated sides were photographed using a microscope at a magnification of 400×. The scale was recorded, and the area of the collateral artery was then quantitated as previously described (31).

### Matrigel plug assay.

The Matrigel plug assay is widely used to assess the angiogenic potential or antiangiogenic activity of different compounds in vivo (63). Briefly, each mouse was injected with 500 μL Matrigel solution (Corning), in which 100 ng/mL VEGF (PeproTech) was added. PRMT5 inhibitor EPZ015666 (the final concentration was 10 μm; Cayman Chemical) or vehicle 0.1% DMSO (Sigma-Aldrich) was added according to different groups. Mice were anesthetized with isoflurane inhalation, and Matrigel containing VEGF, EPZ015666, or DMSO was injected s.c. into the dorsal side of mice. Fifteen days after inoculation, mice were euthanized, and Matrigel plugs were removed. Subsequently, sections were frozen and immunofluorescent staining was performed using anti-CD31 (1:500, 553369, BD Biosciences) antibody and DAPI. Photographs were taken under a confocal microscope. The images were evaluated based on the number or area of new blood vessels.

### Aortic ring sprouting assay.

An aortic ring assay is a simple and effective method to detect new vessels sprouting (64). Briefly, the thoracic aorta segment was carefully separated and divided into a uniform ring approximately 0.5 mm wide. Aortic rings were then inoculated into a 96-well plate containing 40 μL Matrigel/well, and another 40 μL Matrigel was added to cover each ring. In total, 100 μL complete ECM was then added, and VEGF-A was added to the final concentration of 100 ng/mL in the presence and absence of PRMT5 inhibitor EPZ015666. The medium was then changed every other day, and photographs were taken under a microscope during the 5–7 days.

### Lentivirus infection.

Gene knockdown in ECs was performed by lentivirus transduction. pLKO-PRMT5 constructs (TRCN0000107086) and Non-Target shRNA control (SHC016) were from Sigma-Aldrich. Lentiviral pLKO constructs were transfected with packaging and envelope plasmids to HEK293T cells. Viral supernatant was harvested at 72 hours after transfection, filtered through a 0.45 μm filter, and then added to the cultured cells in the media supplemented with 10 μg/mL polybrene (Sigma-Aldrich). Twenty-four hours after infection, the virus-containing media were removed and replaced with fresh media. Knockdown efficiency was detected after infection of virus for 3 days.

### Tube formation assay.

Tube formation assay was performed as previously described (65). In total, 40 μL Matrigel was added into 96-well plates and incubated at 37°C for 20 minutes. HUVECs were counted and diluted into a solution containing 1.5 × 10^5^ cells per milliliter. Mixed cell suspension (100 μL; about 15,000 cells) was seeded on the Matrigel in 96-well plates and incubated from 4 to 16 hours. The images were captured using the EVOS cell imaging system, and the vascular network was quantified using the ImageJ software (NIH) with the angiogenesis analyzer plug-in.

### Wound healing assay.

The wound healing assay is a simple and economical method to study cell migration (66). HUVECs were seeded into 6-well plates and a linear wound was scraped in the center of monolayer cells with a sterile 200 μL pipet suction head; it was then washed 3 times with PBS to remove cell debris. HUVECs migration to the wound was determined at different time points after images were captured using the EVOS FL automated cell imaging system. Migrated cells were quantified using the ImageJ software program.

### CCK-8 assay.

ECs at a density of 70% were digested with trypsin and counted. A total of 1000 cells/well were added into 96-well plates and then incubated with different concentrations of PRMT5 inhibitor EPZ015666 or control vehicle. Six replicates were set at each concentration. In total, 10 μL of WST-8 and 1-methoxyphenazine methyl sulfate (final concentrations of 0.5 mM and 20 μM, respectively) were added to each well and incubated at 37°C for 2–3 hours. The absorbance was measured at 450 nm.

### NO measurement.

To detect endothelial NO production, cell culture supernatants were freshly collected and kept on ice until measurement. The levels of nitrite (NO_2_^–^) and nitrate (NO_3_^–^), the major oxidation product of NO, were determined using a standard calibration curve of potassium iodide detected by chemiluminescence NO detector (Sievers Nitric Oxide Analyzer, NOA 280i, GE Analytical Instruments).

### ELISA.

VEGF-A levels in cell culture supernatants were detected using a human ELISA kit (DVE00, R&D Systems), according to the manufacturer’s instructions. Briefly, collected cell culture supernatants and standards were added in various dilutions to 96-well plates coated with capture antibody, and they were then incubated with biotinylated detection antibody. After incubation with streptavidin-peroxidase and TMB substrate, the protein contents in supernatants were determined by measuring the absorbance at 450 nm (OD_450_) using a plate reader (BioTek Instruments).

### Statistics.

Data are presented as mean ± SD. Student’s *t* test or Mann-Whitney *U* test was applied for comparison between 2 groups. One-way or 2-way ANOVA was performed using GraphPad Prism, version 9, software for comparisons over 2 groups. Post hoc statistical analyses were performed using the Tukey multiple-comparison test. Significance was considered when the *P* value was less than 0.05.

### Study approval.

Animal protocols involving animal care and use were approved by the IACUC at Thomas Jefferson University before initiation of any studies.

## Author contributions

QY, JZ, CZ, BY, KK, WL, XS, and YL performed experiments. QY, JZ, CZ, and JS designed experiments and analyzed and interpreted data. QY, CZ, and JS wrote the paper. All authors edited the paper. First-authorship order was assigned according to the relative amount of data generated in the project in agreement with the 3 co–first authors (QY, JZ, and CZ).

## Supplementary Material

Supplemental data

## Version 1 05/09/2022

Electronic publication
