# Supplementary material for: Endothelial PRMT5 plays a crucial role in angiogenesis after acute ischemic injury
Source: JCI Insight. 2022 May 9;7(9):e152481. doi: 10.1172/jci.insight.152481 (PMC9090242; doi:10.1172/jci.insight.152481)
Supplement: Supplemental data [file jciinsight-7-152481-s267.pdf]

Supplemental material

Supplemental Table 1. Tarlov functional score of ischemic and non-ischemic hindlimb

Score	Function
0	No movement
1	Barely perceptible movement, no weight bearing
2	Frequent and vigorous movement, no weight bearing
3	Supports weight, may take 1 or 2 steps
4	Walks with only mild deficit
5	Normal but slow walking
6	Full and fast walking

Supplemental Figure 1

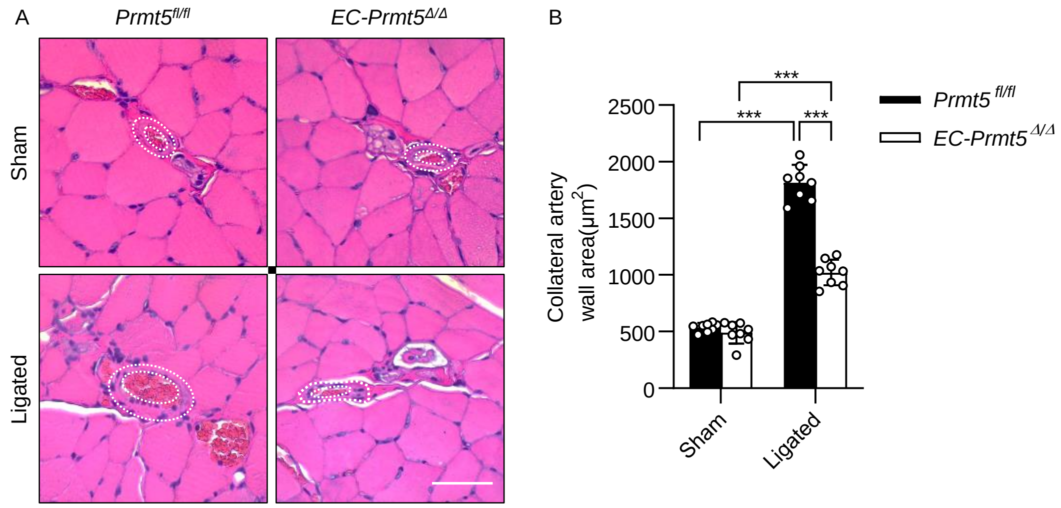

Supplemental Figure 1. Impairment of the femoral collateral arteries dilation in semimembranosus muscles after hindlimb ischemia in EC-specific *Prmt5*-KO mice. **A**. H&E staining of semimembranosus muscles in the ischemic and non-ischemic hindlimbs. The femoral collateral arteries were circumscribed by a dashed white line, and the figures were representative of eight individual mice. Scale bar, 50 μm, n=8. **B**. Quantification of femoral collateral artery wall areas. ****P*<0.001, two-way ANOVA coupled with Tukey's multiple-comparison post hoc test was used. Data were representative of mean ± SD, n=8.

Supplemental Figure 2

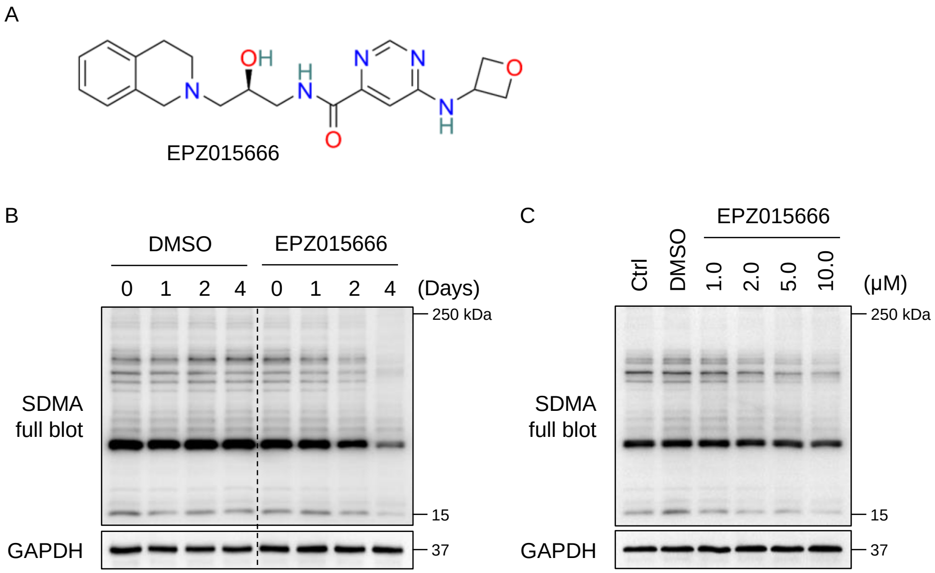

Supplemental Figure 2. Inhibition of PRMT5 enzymatic activity by EPZ015666 in endothelial cells. A, The 2D chemical formula of PRMT5 inhibitor EPZ015666, containing an epoxy quaternion ring. **B,** HUVECs were treated with 10.0 μM EPZ015666 or equal volume of 0.1% DMSO in complete ECM for 0, 2, and 4 days. The expression of SDMA levels was determined by western blot. **C**, HUVECs at 70%-80% confluence were treated with 0.1% DMSO or indicated concentrations of EPZ015666 for 4 days. The levels of SDMA were determined by western blot.
